# Solid Pseudopapillary Neoplasm of the Pancreas: Diagnostic Concordance and Clinicopathologic Correlation of Fine-Needle Aspiration and Long-Term Outcomes

**DOI:** 10.7759/cureus.113100

**Published:** 2026-07-21

**Authors:** Pegah Dejban, Eric Z Wang, Beena U Ahsan, Lisi Yuan

**Affiliations:** 1 Department of Pathology and Laboratory Medicine, Henry Ford Health System, Detroit, USA; 2 Readying Youth Scientists for Excellence in Medicine, Education and Discovery (RYSE MED) Program, Henry Ford Hospital, Troy, USA; 3 College of Human Medicine, Michigan State University, East Lansing, USA

**Keywords:** cytology, fine-needle aspiration, long-term outcomes, pancrea, solid pseudopapillary neoplasm, β-catenin

## Abstract

Introduction: Solid pseudopapillary neoplasm (SPN) is a rare pancreatic tumor with low malignant potential and unclear cell of origin and pathogenesis. Fine-needle aspiration (FNA) is frequently used for preoperative diagnosis, yet correlation with resection specimens and long-term follow-up remains limited in the literature.

Materials and methods: We retrospectively identified all patients diagnosed with SPN by pancreatic FNA at a single institution (2016-2025). Cytology preparations included smears with or without rapid on-site evaluation and ancillary immunohistochemistry performed on cell blocks when available. Surgical pathology, when performed, was reviewed for diagnostic concordance and adverse features. Clinical outcomes were assessed by chart review.

Results: Seven patients were identified (female-to-male ratio = 6:1; median age = 29 years, range = 18-53 years). Tumors most commonly involved the tail (5/7) and measured a median of 6.0 cm (range = 2.6-14.0). Pancreatic FNA rendered a definitive diagnosis of SPN in six cases (6/7). Six patients underwent resection, and surgical pathology confirmed SPN in all resected tumors (6/6). Lymphovascular invasion was absent in all resected cases; perineural invasion was present in one case. At last follow-up (median = 41 months, range = 4-119), all patients were alive. One patient developed liver metastasis eight years after the initial diagnosis.

Conclusions: Pancreatic FNA demonstrated excellent diagnostic concordance and clinicopathologic correlation for SPN with available resections. Despite overall indolent behavior, late metastatic disease can occur, supporting the need for long-term surveillance.

## Introduction

Solid pseudopapillary neoplasm (SPN) is an uncommon pancreatic epithelial neoplasm that represents 1-2% of pancreatic neoplasms, typically affecting young women and often arising in the pancreatic body or tail. Although most SPNs behave indolently, a subset demonstrates local invasion, recurrence, or metastasis, and delayed relapses have been documented [1-3].

Preoperative diagnosis has become increasingly important as surgical approaches evolve toward organ-preserving strategies and as pancreatic masses in young patients encompass a broad differential. Endoscopic ultrasound-guided fine-needle aspiration (EUS-FNA) is widely used for tissue acquisition and can reliably render a specific diagnosis when characteristic cytomorphology is recognized with the assistance of ancillary testing [4-6].

Cytologically, SPN often shows delicate papillary fronds, loosely cohesive monomorphic cells with finely granular chromatin, inconspicuous nucleoli, characteristic longitudinal nuclear grooves, and cytoplasmic vacuolation; eosinophilic hyaline globules and foamy macrophages may be present. Immunohistochemistry is frequently helpful in limited samples, with nuclear β-catenin reflecting canonical Wnt pathway activation and frequent CTNNB1 alterations [7-10].

Given the rarity of SPN, many institutional cytology practices encounter these tumors infrequently. We therefore evaluated the diagnostic concordance and clinicopathologic correlation of fine-needle aspiration (FNA) for SPN in a contemporary single-institution cohort, correlated cytologic diagnoses with available resections, and summarized long-term outcomes, including one case with late liver metastasis.

## Materials and methods

This retrospective, single-institution study was conducted at Henry Ford Health in Detroit, Michigan. The institutional cytopathology database was searched to identify patients who underwent EUS-FNA of a pancreatic mass during the initial diagnostic evaluation and were confirmed to have SPN by cytologic evaluation and/or subsequent surgical pathology between May 2016 and April 2025. A nondiagnostic EUS-FNA did not preclude inclusion when SPN was subsequently confirmed on surgical resection. Patients presenting with recurrent disease, those without an initial pancreatic EUS-FNA, and those with metastatic disease at presentation were excluded. The liver FNA obtained eight years after the initial pancreatic diagnosis in case 5 was reviewed solely for clinicopathologic correlation and was not used to determine eligibility for inclusion. Clinical, radiologic, and follow-up data were obtained through review of the electronic medical record, with clinical follow-up available through April 2026.

Pancreatic specimens were obtained by EUS-FNA. Air-dried smears were stained with Diff-Quik, whereas alcohol-fixed smears were prepared using the Papanicolaou method; rapid on-site evaluation (ROSE) was performed when applicable. Available cytology smears and cell block preparations were reviewed to evaluate the characteristic cytomorphologic features of SPN. Immunohistochemistry was performed on cell blocks and resection specimens when indicated. A focused panel emphasizing nuclear β-catenin, along with markers commonly used in the differential diagnosis (e.g., neuroendocrine markers, acinar cell carcinoma markers, and epithelial markers), was used to support SPN when needed.

For patients who underwent surgical resection, the corresponding histopathologic slides were reviewed to confirm the diagnosis and assess adverse features, including lymphovascular invasion (LVI), perineural invasion (PNI), tumor necrosis, and the presence of an undifferentiated component. Pathologic stage was obtained from the corresponding synoptic pathology reports when available. Given the small cohort size and descriptive nature of the study, analyses were limited to descriptive statistics, and no formal hypothesis testing was performed.

## Results

A total of seven patients met the study inclusion criteria, and their demographic, clinical, radiologic, cytologic, and histopathologic characteristics are summarized in Table 1.

**Table 1:** Demographics, clinicopathological features, and cyto-histo correlation of solid pseudopapillary neoplasm of the pancreas. * Pancreatic cyst fluid carcinoembryonic antigen (CEA) level (patient 2); all others: serum CEA level. ** This patient had a lung nodule four months after diagnosis of a solid pseudopapillary neoplasm, which turned out to be sarcoidosis. *** This patient had liver metastasis after eight years of diagnosis.

	Patient 1	Patient 2	Patient 3	Patient 4	Patient 5	Patient 6	Patient 7
Sex	Female	Female	Female	Male	Female	Female	Female
Age at diagnosis (years)	53	26	18	28	29	29	26
Survival status	Alive	Alive	Alive	Alive	Alive	Alive	Alive
Significant past medical history	No	No	No	No	No	No	No
Alcohol consumption	Yes	Yes	Yes	Yes	Yes	Yes	Yes
Smoking history	No	No	No	No	No	No	Yes
Family history	No	No	No	No	No	No	No
Initial symptoms	Sudden onset left-sided abdominal pain	Sudden onset left-sided abdominal pain with nausea and vomiting	Constant left-sided abdominal pain radiating to the back	Nonspecific abdominal discomfort after drinking alcohol or eating a large meal	Cramping and stabbing pain in the left upper quadrant	Vague epigastric pain with nausea	Sudden onset right upper quadrant pain
Time from onset of symptom to diagnosis (day)	3	5	7	28	10	60	3
CT/MRI findings	Heterogeneous enhancing mass	Cyst with internal septation	Mixed solid and cystic mass	Multilobulated solid-cystic mass with central irregular necrosis	Heterogeneous enhancing mass	Solid cystic mass in	Solid, partially exophytic pancreatic mass
Carcinoembryonic antigen (CEA) (ng/ml)	2.7	0.8*	0.2	Not evaluated	<0.5	<0.5	0.8
Serum amylase (IU/L)	41	16	549	279	5	47	129
Fine-needle aspiration diagnosis	Solid pseudopapillary	Non-diagnostic (cyst contents)	Solid pseudopapillary	Solid pseudopapillary	Solid pseudopapillary	Solid pseudopapillary	Solid pseudopapillary
Beta-catenin immunohistochemistry (cell block)	Nuclear stain	Not applicable	Nuclear stain	Nuclear stain	Nuclear stain	Nuclear stain	Nuclear stain
Surgical pathology diagnosis	No surgery	Solid pseudopapillary	Solid pseudopapillary	Solid pseudopapillary	Solid pseudopapillary	Solid pseudopapillary	Solid pseudopapillary
Tumor size (cm)	2.6	6	14	7	11.8	6	6
Tumor location	Pancreas head	Pancreas tail	Pancreas tail	Pancreas tail	Pancreas tail	Pancreas tail	Pancreas head
Tumor stage	No surgery	pT3N0M0	pT3N0M0	pT3N0M0	pT3N0M0	pT3N0M0	pT3N0M0
Lymphovascular invasion (LVI)	No surgery	No	No	No	No	No	No
Perineural invasion (PNI)	No surgery	No	No	No	Yes	No	No
Local recurrence/distant metastasis	No	No	No	No**	Yes***	No	No
Interval between diagnosis and last follow-up (months)	38	41	22	4	119	79	42

Six patients were females, and one was a male, corresponding to a female-to-male ratio of 6:1. The median age at diagnosis was 29 years (range = 18-53 years). All patients presented with abdominal pain or discomfort, with a median symptom duration of 10 days before clinical evaluation (range = 3-60 days). Tumors were located in the pancreatic tail in five patients and in the pancreatic head in two patients. Tumor size ranged from 2.6 to 14.0 cm, with a median diameter of 6.0 cm.

**Figure 1:**
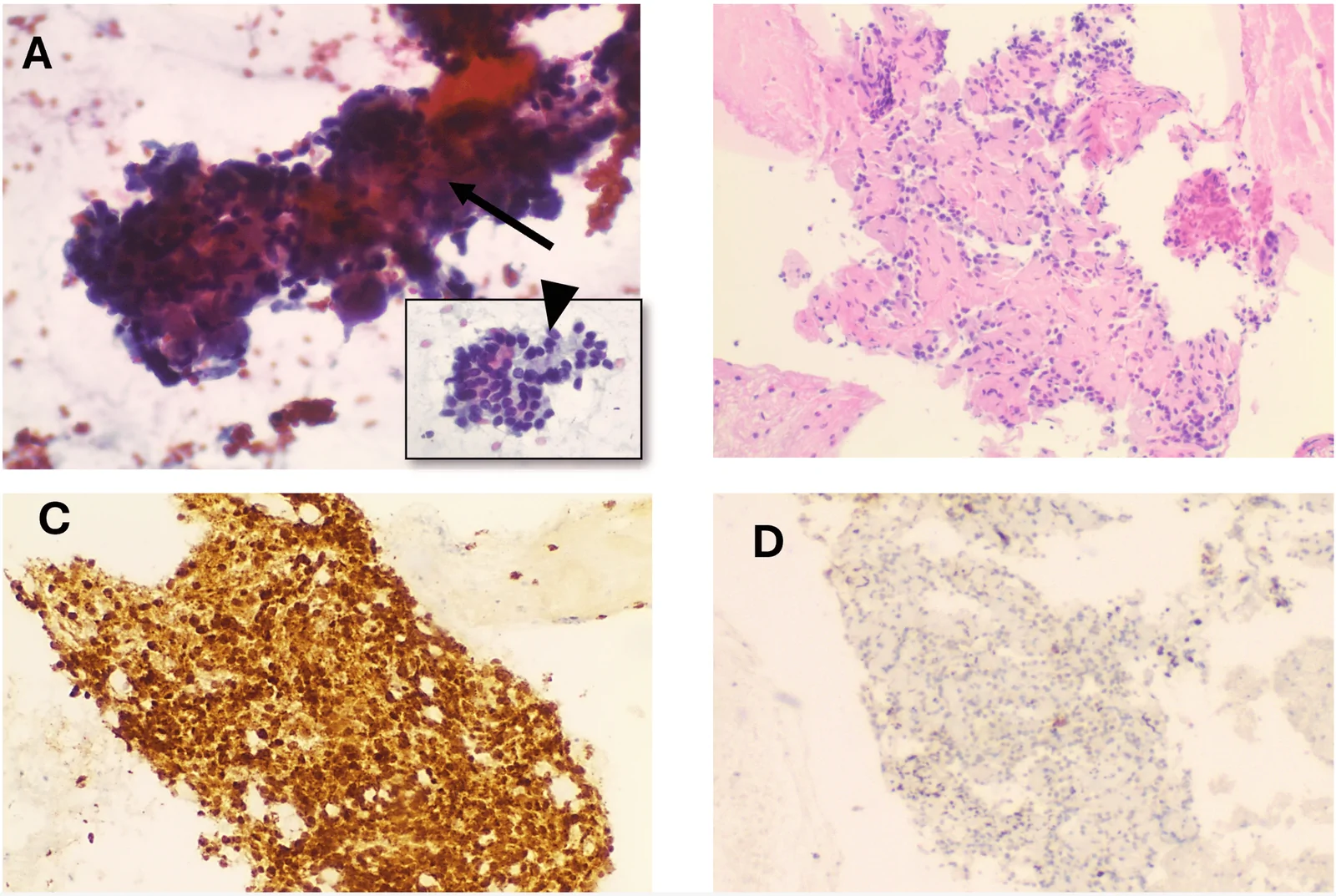
Representative cytologic and immunohistochemical findings of case 1. (A) Diff-Quik smear showing pseudopapillae or rosette-like structures formed due to tumor cells getting detached from delicate, branching vessels (arrow); inset, higher magnification of loosely cohesive bland monomorphic cells with finely granular chromatin and eosinophilic cytoplasm (arrowheads). Papanicolaou stain, 200x. (B) Cell block showing characteristic pseudopapillary architecture (arrow) (H&E, 100x). (C) Diffuse nuclear β-catenin immunoreactivity (200x). (D) Negative synaptophysin immunostaining (100x).

EUS-FNA established a definitive diagnosis of SPN in six of the seven patients. In the remaining patient, the aspirate consisted predominantly of cyst contents and was considered nondiagnostic; subsequent surgical resection confirmed SPN. ROSE was performed in four cases and was interpreted as adequate in three and indeterminate in one. One patient received a definitive diagnosis based on the characteristic cytomorphologic findings and supportive immunophenotype but did not undergo surgical resection because of multiple comorbidities (case 1); the corresponding cytologic and immunohistochemical findings are shown in Figure 1.

**Figure 2:**
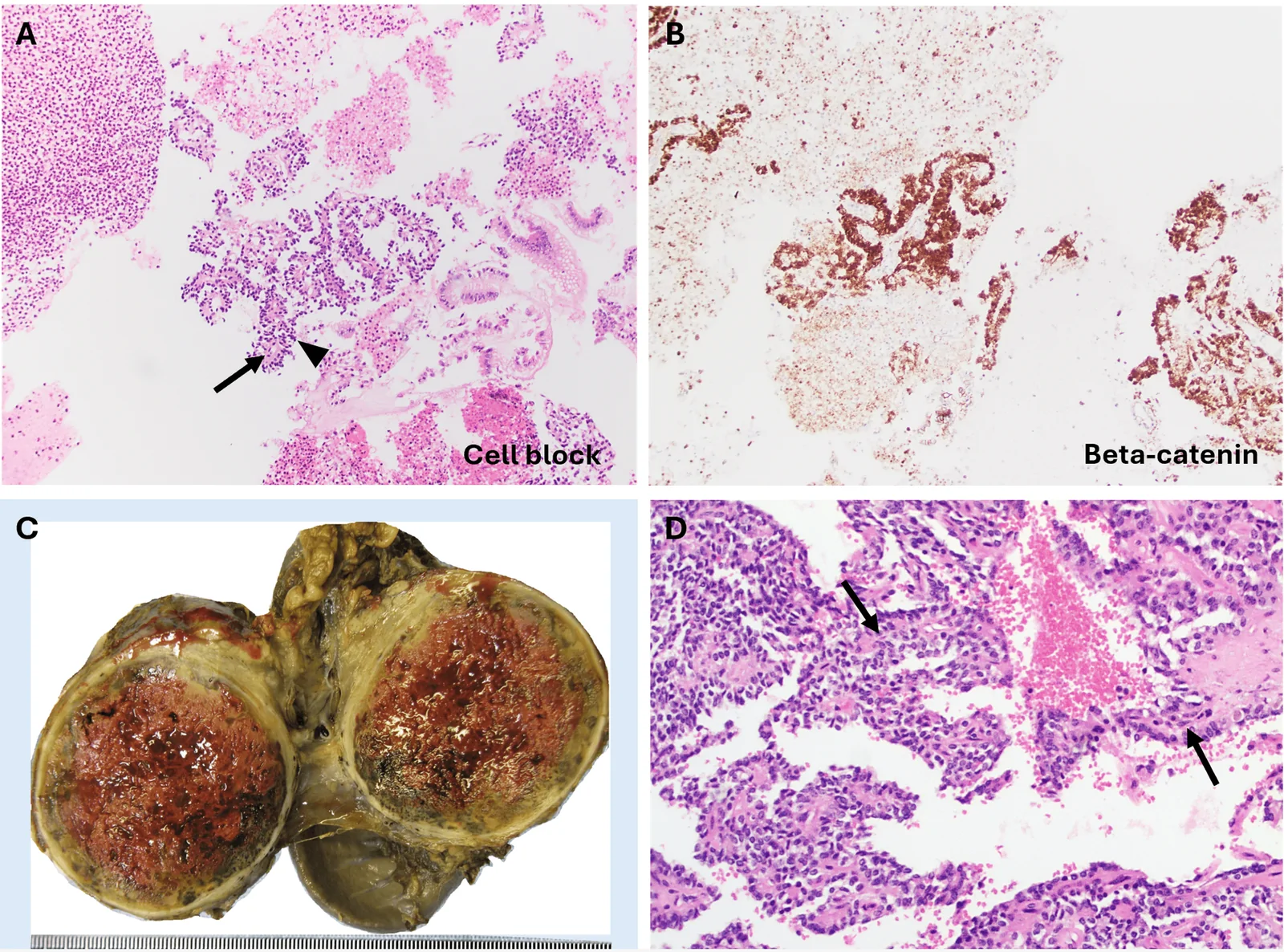
Representative cytologic, immunohistochemical, gross, and histologic findings of pancreatic solid pseudopapillary neoplasm (case 2). (A) Cell block demonstrating characteristic pseudopapillary architecture with delicate branching vessels (arrow) lined by loosely cohesive, uniform tumor cells (arrowheads) with round to oval nuclei, finely dispersed chromatin, inconspicuous nucleoli, and occasional nuclear grooves (H&E, ×40). (B) Diffuse nuclear β-catenin immunoreactivity (×40). (C) Gross examination showing a well-circumscribed solid and cystic pancreatic mass with areas of hemorrhagic degeneration. (D) Surgical resection showing characteristic solid and pseudopapillary architecture with uniform neoplastic cells surrounding delicate branching vessels (arrow) (H&E, ×40).

Six patients underwent surgical resection, and all six resection specimens were histologically confirmed as SPN. Among the five resected patients who had received a definitive cytologic diagnosis, FNA and surgical pathology diagnoses were concordant in all cases (5/5). The nondiagnostic FNA (case 2) and its subsequent resection findings are illustrated in Figure 2.

**Figure 3:**
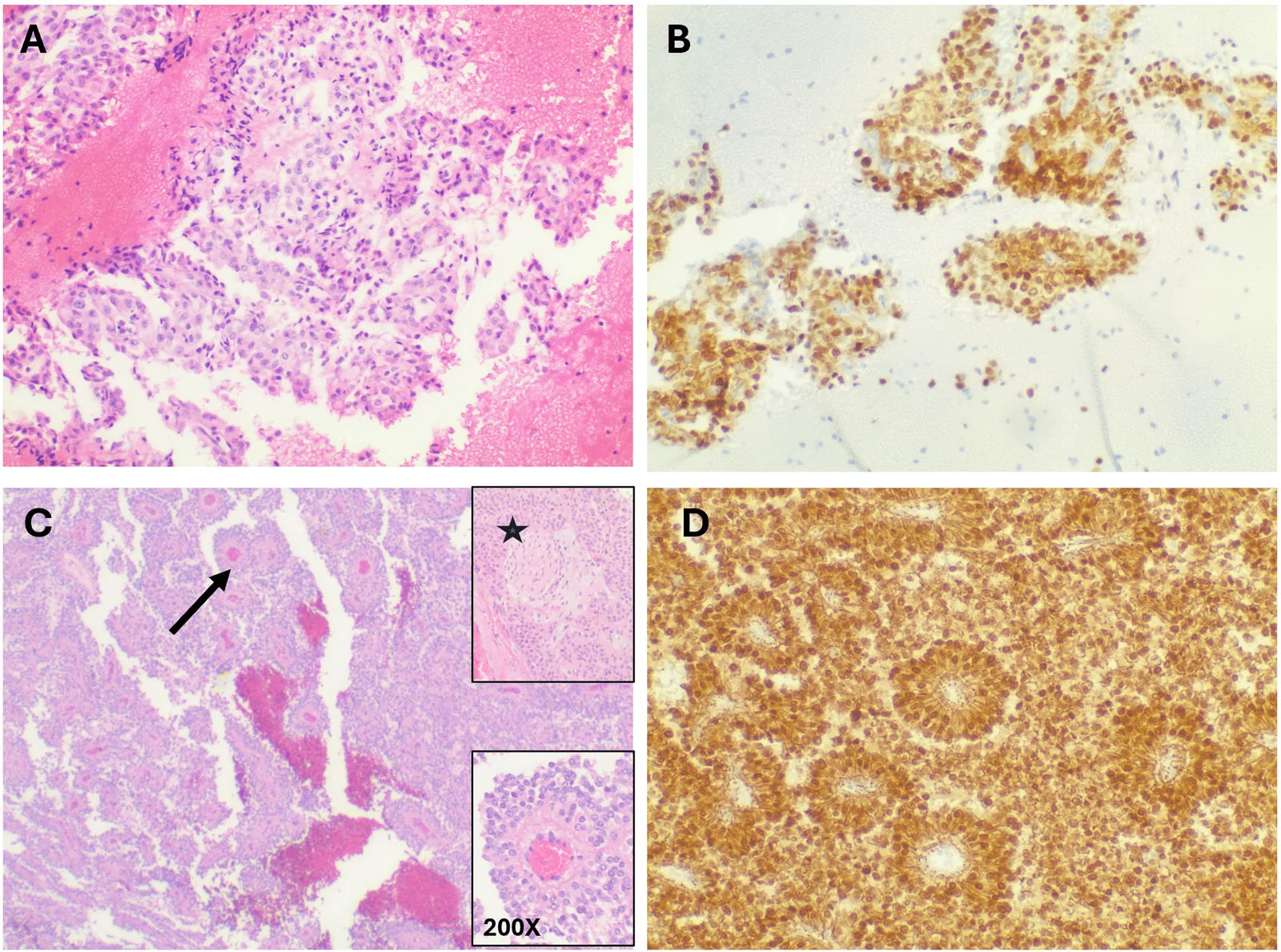
Representative cytologic, immunohistochemical, and histologic findings of metastatic pancreatic solid pseudopapillary neoplasm (case 5). (A) Liver fine-needle aspiration (FNA) cell block demonstrating characteristic pseudopapillary architecture with delicate fibrovascular cores (arrow) lined by loosely cohesive, uniform tumor cells (arrowheads) exhibiting round to oval nuclei, finely dispersed chromatin, inconspicuous nucleoli, and occasional nuclear grooves (H&E, ×100). (B) Diffuse nuclear β-catenin immunoreactivity (×100). (C) Pancreatic resection showing characteristic solid and pseudopapillary architecture (arrow) with perineural invasion (*); upper inset showing higher magnification of the involved nerve, lower inset showing higher magnification of a pseudopapilla (H&E, ×20; inset ×200). (D) Diffuse nuclear β-catenin immunoreactivity in the pancreatic tumor (×100).

Within the resected cohort, lymphovascular invasion, tumor necrosis, and an undifferentiated component were absent in all tumors, whereas focal perineural invasion was identified in one patient (case 5). Representative findings from this case are presented in Figure 3.

Clinical follow-up ranged from four to 119 months, with a median follow-up of 41 months. All patients were alive at the most recent follow-up (April 2026). Five patients had no evidence of local recurrence or distant metastasis. Case 5 developed liver metastasis eight years after the initial pancreatic EUS-FNA diagnosis; the metastatic lesion was subsequently diagnosed by liver FNA and confirmed by segmental hepatic resection. The liver FNA was performed during the follow-up period and was not used as the basis for inclusion in the study. Another patient developed a pulmonary nodule four months after diagnosis; however, subsequent evaluation established a diagnosis of sarcoidosis rather than metastatic SPN.

## Discussion

In this single-institution retrospective cohort, pancreatic EUS-FNA demonstrated strong diagnostic concordance and clinicopathologic correlation for SPN, with a definitive cytologic diagnosis rendered in six of seven cases. Among the resected cases with a definitive cytologic diagnosis, there was complete concordance with the surgical pathology findings. These results are consistent with previous reports indicating that EUS-FNA is well suited for the diagnosis of SPN in limited specimens when the characteristic architecture and cytomorphologic features are recognized, and adequate cell block material is available for ancillary testing [4-6]. Although one patient did not undergo surgical resection because of multiple comorbidities, the diagnosis of SPN was considered definitive based on the characteristic cytomorphologic features and supportive immunophenotype, including diffuse nuclear β-catenin expression.

An important diagnostic challenge in pancreatic cytology is the morphologic overlap between SPN and other neoplasms, particularly pancreatic neuroendocrine tumor (PanNET), acinar cell carcinoma, pancreatoblastoma, and cystic pancreatic lesions. In this setting, ancillary immunohistochemistry can be particularly valuable. Nuclear localization of β-catenin, which reflects activation of the Wnt signaling pathway and frequent CTNNB1 alterations, is a characteristic feature of SPN and can be demonstrated in cell block preparations [9,11]. In contrast, PanNET typically shows diffuse chromogranin and synaptophysin positivity and lacks nuclear β-catenin [12], whereas acinar cell carcinoma demonstrates positivity for pancreatic enzymes (trypsin, chymotrypsin) and BCL10 [13,14]. Although the majority of pancreatoblastoma is positive for beta-catenin as well (approximately 80%), this tumor is more common in young children, contains multilineage components, and forms squamoid nests [15]. In addition, SPN frequently undergoes cystic degeneration, especially in larger tumors [7]. Other pancreatic cystic lesions, particularly mucinous cystic neoplasm (MCN), are also commonly seen in females and involve the distal pancreas. However, MCN usually presents in the fourth to fifth decade of life (mean age of 45 years). Elevated carcinoembryonic antigen (CEA) level, presence of mucin/mucinous epithelium, and presence of KRAS mutation in cyst fluid support a mucinous cyst, while cyst fluid chemistry shows low CEA and amylase in SPN [16]. Differential diagnosis is summarized in Table 2.

**Table 2:** Differential diagnosis of solid pseudopapillary neoplasm based on cytological features. LEF1: lymphoid enhancer-binding factor-1; AR: androgen receptor; PR: progesterone receptor; INSM1: insulinoma-associated protein 1; BCL10: B-cell lymphoma/leukemia 10. APC gene: Adenomatous polyposis coli gene; germline mutation leads to familial adenomatous polyposis. MEN1 gene: Multiple endocrine neoplasia type 1 gene; germline mutation leads to multiple endocrine neoplasia type 1.

Diagnosis	Smear cellularity	Pattern	Cells	Nuclear details	Cytoplasmic details	Immunohistochemistry	Molecular
Solid pseudopapillary neoplasm	Moderate to high	Delicate papillary fronts	Bland, uniform cells with discohesion	Round to oval with longitudinal grooves, finely granular chromatin, and inconspicuous nucleoli	Moderate eosinophilic cytoplasm with perinuclear vacuoles, eosinophilic hyaline globules	Beta-catenin (nuclear), E-cadherin (loss), alpha-1-antitrypsin (hyaline globules), cyclin D1/ LEF1, AR/PR	A point mutation in exon 3 of the β-catenin gene (CTNNB1) was present in > 90%. Rare mutations in the APC gene [11].
Neuroendocrine tumor	Moderate to high	Small groups and single cells	Small, uniform, dispersed cells	Eccentric nuclei, salt and pepper chromatin, no evident nucleoli	Moderate eosinophilic granular cytoplasm	Synaptophysin, chromogranin, INSM1, keratins	MEN1 inactivation in 44%, DAXX/ATRX mutation or loss in 43%, mTOR pathway alterations in 11% [12].
Acinar cell carcinoma	Moderate to high	Small groups and single cells	Small, uniform cells	Monomorphic nuclei with prominent nucleoli	Finely granular eosinophilic cytoplasm	Keratins, pancreatic enzymes (trypsin, chymotrypsin), BCL10. Beta-catenin (nuclear) in 10%.	BRAF, RAF1, and RET rearrangements in one-third [13,14].
Pancreatoblastoma	Variable	Cohesive groups, squamoid nests	Multilineage, squamoid cells	Multilineage, high-grade, may include cells with acinar, neuroendocrine, or ductal differentiation, and primitive cells	Multilineage, primitive cells have minimal cytoplasm and a high nuclear-to-cytoplasmic ratio	Beta-catenin (nuclear) in squamoid nest.	Allelic loss on chromosome 11p; molecular alterations in the APC/beta-catenin pathway [15].
Mucinous cystic neoplasm	Low	Strips of mucinous epithelium	Intestinal or gastric type	Variable depending on the type of epithelium and degree of dysplasia	Intracytoplasmic mucin	Keratins in epithelium. ER and inhibin in ovarian type stoma.	KRAS mutations [16]

The clinicopathologic findings in our cohort correlate with the established profile of SPN, including a marked female predominance and frequent involvement of the pancreatic tail [1-3]. All patients were alive at the most recent follow-up (April 2026), supporting the generally favorable prognosis associated with this neoplasm. Nevertheless, the development of liver metastasis eight years after the initial diagnosis demonstrates that SPN, although usually indolent, retains the potential for delayed metastatic progression. Larger clinicopathologic studies have similarly documented a low but clinically meaningful risk of recurrence or metastasis and have suggested that tumor size and adverse pathologic findings, such as LVI and an elevated proliferative index, may be associated with increased risk [17,18]. Notably, all our resected cases were staged as pT3 but lacked LVI, with only one case showing focal PNI. This case later developed liver metastasis, suggesting that PNI may also be an adverse feature to predict the risk for recurrence or metastatic disease.

Accurate preoperative recognition of SPN by EUS-FNA may facilitate appropriate clinical and surgical planning. Establishing a specific diagnosis is particularly important in younger patients because it can help distinguish SPN from more aggressive pancreatic neoplasms and support a tailored surgical strategy [2,4]. Moreover, recognition of the characteristic cytomorphologic features, together with appropriate ancillary testing, may allow a definitive diagnosis even when surgery is not feasible in certain situations and surgical pathology correlation will not be available.

The principal limitations of this study include the small sample size, which reflects the rarity of SPN, as well as its retrospective, single-institution design. The study was also descriptive and did not include formal statistical modeling. In addition, the immunohistochemical panels and procedural details varied according to specimen availability, clinical context, and operator practice during the study period. Future multi-institutional studies incorporating standardized cytologic-histologic correlation, uniform ancillary testing, and extended clinical follow-up may provide further insight into the factors associated with recurrence and metastatic progression.

## Conclusions

Pancreatic EUS-FNA is a valuable diagnostic approach for SPN, particularly when characteristic cytomorphologic features are interpreted in conjunction with supportive immunohistochemical findings, including nuclear β-catenin expression. In this cohort, definitive cytologic diagnoses showed excellent concordance with available surgical pathology specimens, supporting the role of EUS-FNA in the preoperative evaluation of these tumors. Although SPN generally follows an indolent clinical course and is associated with a favorable prognosis, the case of the development of liver metastasis eight years after the initial diagnosis highlights the potential for delayed disease progression. Therefore, long-term clinical surveillance should be considered even for patients with apparently low-risk tumors and no evidence of recurrence during early follow-up.
